# Two novel variants in *CNNM2* disrupts magnesium efflux leading to neurodevelopmental disorders

**DOI:** 10.3389/fgene.2025.1600877

**Published:** 2025-06-19

**Authors:** Huijuan Li, Jing Liu, Yingdi Liu, Yaning Liu, Kehui Lu, Juan Wen, Huimin Zhu, Desheng Liang, Zhuo Li, Lingqian Wu

**Affiliations:** ^1^ MOE Key Lab of Rare Pediatric Diseases, Center for Medical Genetics, Hunan Key Laboratory of Medical Genetics, School of Life Sciences, Central South University, Changsha, China; ^2^ Hunan Provincial Key Laboratory of Regional Hereditary Birth Defects Prevention and Control, Changsha Hospital for Maternal and Child Healthcare, Hunan Normal University, Changsha, China; ^3^ Department of Medical Genetics, Hunan Jiahui Genetics Hospital, Changsha, China; ^4^ Hainan Provincial Key Laboratory for Human Reproductive Medicine and Genetic Research, Department of Reproductive Medicine, Hainan Provincial Clinical Research Center for Thalassemia, Key Laboratory of Reproductive Health Diseases Research and Translation, Hainan Medical University, Haikou, Hainan, China; ^5^ Department of medical Artificial Intelligence, Bright prosperity institute, Hangzhou, China

**Keywords:** hypomagnesemia, seizures, intellectual disability, whole exome sequencing, CNNM2

## Abstract

**Background:**

Hypomagnesemia, seizures, and impaired intellectual development 1 (HOMGSMR1) is a rare neurodevelopmental disorder associated with magnesium homeostasis disruption, caused by mutations in the *CNNM2* gene. HOMGSMR1 demonstrates considerable clinical heterogeneity, but the genotype-phenotype relationship remains insufficient.

**Methods:**

We recruited two unrelated families with NDDs, and potential variants were identified through whole exome sequencing and confirmed by Sanger sequencing. Quantitative PCR, Western blotting, immunofluorescent staining, and flow cytometry were used to assess functional changes in candidate *CNNM2* variants.

**Results:**

Two novel variants, p.E298del and p.P360R, in *CNNM2* gene were identified. The unique facial features of proband 1 may broaden the known phenotypic spectrum of HOMGSMR1. Functional studies confirmed that the p.E298del and p.P360R variants increased *CNNM2* transcription and protein levels, impairing the proper localization of the CNNM2 protein to the cell membrane. Two variant proteins accumulated in the cytoplasm and formed clumps. Furthermore, intracellular Mg^2+^ levels were higher in cells with these variants, disrupting magnesium homeostasis and potentially contributing to hypomagnesemia. Notably, the proteins of these two variants exhibited reduced stability and were prone to degradation, potentially providing new insights into the pathogenic mechanisms of *CNNM2*.

**Conclusion:**

Our study expands the mutation and phenotypic spectrum, as well as the functional studies of *CNNM2*, and contributes to genetic testing and prenatal diagnosis in families with HOMGSMR1.

## 1 Introduction

Neurodevelopmental disorders (NDDs) are a group of diseases that affect brain development and function, characterized by significant genetic and clinical heterogeneity (Thapar et al., 2017; Parenti et al., 2020). NDDs are influenced by complex genetic and non-genetic factors (Srivastava et al., 2019; Han et al., 2021), with 1, 586 high-confidence NDD genes and 6, 478 candidate genes identified (Leblond et al., 2021). However, approximately 60% of patients remain undiagnosed genetically (Srivastava et al., 2019), resulting in a substantial economic and social burdens (Collaborators, 2018). Numerous studies have shown that genes associated with neurodevelopmental disorders are often closely linked to biometal dyshomeostasis (Bourre, 2006; Garza-Lombó et al., 2018; Błażewicz and Grabrucker, 2022). Metals play a crucial role in brain development. Among biometals, magnesium is especially vital, as it is crucial for maintaining neuronal growth and development, myelination, synaptic function, and signal transduction (Xu et al., 2014; Yamanaka et al., 2019). Notably, cyclin M2 (CNNM2) appears to be the first identified regulator of magnesium ion homeostatic factor (Sponder et al., 2016).

The *CNNM2* gene (OMIM *607803) is located on the chromosome 10q24.32, consists of eight exons, and it encodes the CNNM2 protein comprising 875 amino acids (aa) (Wang et al., 2003). CNNM2 protein can be widely expressed in brain, distal convoluted tubule of the kidney, and in lung (Wang et al., 2003). It contains five functional domains such as an N-terminal extracellular domain, four transmembrane domains (also known as DUF21 domain), two cystathionin beta synthase domains (CBS1, CBS2), and a C-terminal cyclic nucleotide-binding homology (CNBH) domain (Wang et al., 2003; de Baaij et al., 2012). Moreover, *CNNM2* was involved in magnesium ion (Mg^2+^) transport, and *CNNM2* mutations could lead to disruptions in magnesium homeostasis (Corral-Rodríguez et al., 2014; Hirata et al., 2014; de Baaij, 2015; Li et al., 2017; Huang et al., 2021). Both homozygous and heterozygous variants in the *CNNM2* gene cause different disease phenotypes. In 2011 (Stuiver et al., 2011), first proposed that heterozygous *CNNM2* gene mutations were associated with renal hypomagnesemia-6 (HOMG6, OMIM #613882). Subsequently (Arjona et al., 2014), identified heterozygous or homozygous *CNNM2* mutations in patients with NDDs, revealing a connection between *CNNM2* gene mutations and hypomagnesemia, seizures, and impaired intellectual development 1 (HOMGSMR1, OMIM #616418). Additionally, they demonstrated that knockdown of *Cnnm2* in zebrafish led to disturbed brain development. Similarly, knockout of the *Cnnm2* in mice resulted in maldevelopment of the brain, notably characterized by exencephaly (Franken et al., 2021b). HOMGSMR1 demonstrated considerable clinical heterogeneity, with affected individuals typically exhibiting intellectual disability, seizures, developmental delay, hypomagnesemia, obesity, and psychiatric or behavioral abnormalities. Furthermore, several studies have reported that *CNNM2* gene is also associated with schizophrenia (Thyme et al., 2019; Liu et al., 2021), hypertension (Funato et al., 2017), intracranial aneurysm (Liu M. et al., 2023; Wu et al., 2024), myocardial infarction (Matsuoka et al., 2015), pulmonary hypertension (Wang et al., 2021), and sleep apnea (Gui et al., 2024), among other conditions.

To date, 37 disease-associated *CNNM2* variants have been reported (Stuiver et al., 2011; Arjona et al., 2014; Accogli et al., 2019; García-Castaño et al., 2020; Bamhraz et al., 2021; Franken et al., 2021a; Huang et al., 2021; Li et al., 2021; Panda et al., 2021; Zhang et al., 2021; Petrakis et al., 2022; Tseng et al., 2022; Xu et al., 2022; Liu C. X. et al., 2023; Wang et al., 2023; Bosman et al., 2024). Of these, 30 variants, involving 41 patients, were related to *CNNM2*-associated NDDs (Arjona et al., 2014; Accogli et al., 2019; Bamhraz et al., 2021; Franken et al., 2021a; Huang et al., 2021; Li et al., 2021; Panda et al., 2021; Zhang et al., 2021; Petrakis et al., 2022; Tseng et al., 2022; Xu et al., 2022; Liu C. X. et al., 2023; Wang et al., 2023; Bosman et al., 2024). Different variant sites exhibited distinct phenotypes and inheritance patterns. Variants in the DUF21 domain were most strongly associated with *CNNM2* related central nervous system phenotypes, while hypomagnesemia was more pronounced in patients with CBS2 domain variants (Zhang et al., 2021). Additionally, autosomal recessive (AR) inherited *CNNM2* related disorders were associated with the most severe phenotype (Arjona et al., 2014; Accogli et al., 2019; Zhang et al., 2021). Given the complexity of the *CNNM2* genotype-phenotype relationship, existing studies still offer a limited understanding of its correlation. Many variants of uncertain significance (VUS) provide limited guidance for family planning, severely compromising the effectiveness of genetic counseling. These patients typically presented with varying degrees of hypomagnesemia, often in the form of refractory hypomagnesemia, which posed significant challenges for clinical management.

In this study, we identified two unrelated HOMGSMR1 patients carrying *CNNM2* variants. Through a comprehensive analysis of the proband’s clinical phenotype, genetic data, and functional experiments, we confirmed the pathogenicity of these two variants, thereby expanding the mutation and phenotypic spectrum of the *CNNM2* gene.

## 2 Materials and methods

### 2.1 Probands

Two unrelated Chinese families with NDDs were recruited through Hunan Jiahui Genetics Hospital. The proband 1 and proband 2 presented with intellectual disability, developmental delay, hypomagnesemia, and abnormal mental behavior. This study obtained informed consent from the guardians of minor patients, who signed the respective consent forms. Additionally, it received approval from the Medical Ethics Committee of Central South University, Hunan, China (No, 202107009, Date: 2021-8-27).

### 2.2 Whole-exome sequencing (WES) and bioinformatics analysis

Genomic DNA from the main members of two families was extracted from peripheral blood using the QuickGene DNA Whole Blood Kit L (FUJIFILM, Tokyo, Japan) according to standard extraction methods. Available DNA was sequenced using WES by Berry Genomics Inc., Beijing, China. The sequencing data used human reference genome version 19 (hg19) as the reference sequence. All variants were analyzed with an allele frequency <5% based on their presence in Exome Aggregation Consortium (ExAC) projects, the Genome Aggregation Database (gnomAD), and the 1000 Genomes Project (1000G). The pathogenicity of the variants was predicted using MutationTaster (http://www.mutationtaster.org/), PolyPhen-2 (http://genetics.bwh.harvard.edu/pph2/), SIFT (http://blocks.fhcrc.org/sift/SIFT.html), REVEL (https://sites.google.com/site/revelgenomics/), and others. All candidate variants were classified according to the recommendations of the American College of Medical Genetics and Genomics (ACMG) (Richards et al., 2015). Conservation analyses were performed using T-coffee software, and amino acid sequence data were sourced from the National Center for Biotechnology Information (NCBI). The three-dimensional structural models of wild type and two mutant CNNM2 proteins were predicted using AlphaFold2 database and visualized with PyMOL software to observe changes in hydrogen bonds between amino acids and protein surface charges.

### 2.3 Sanger sequencing

**FIGURE 1:**
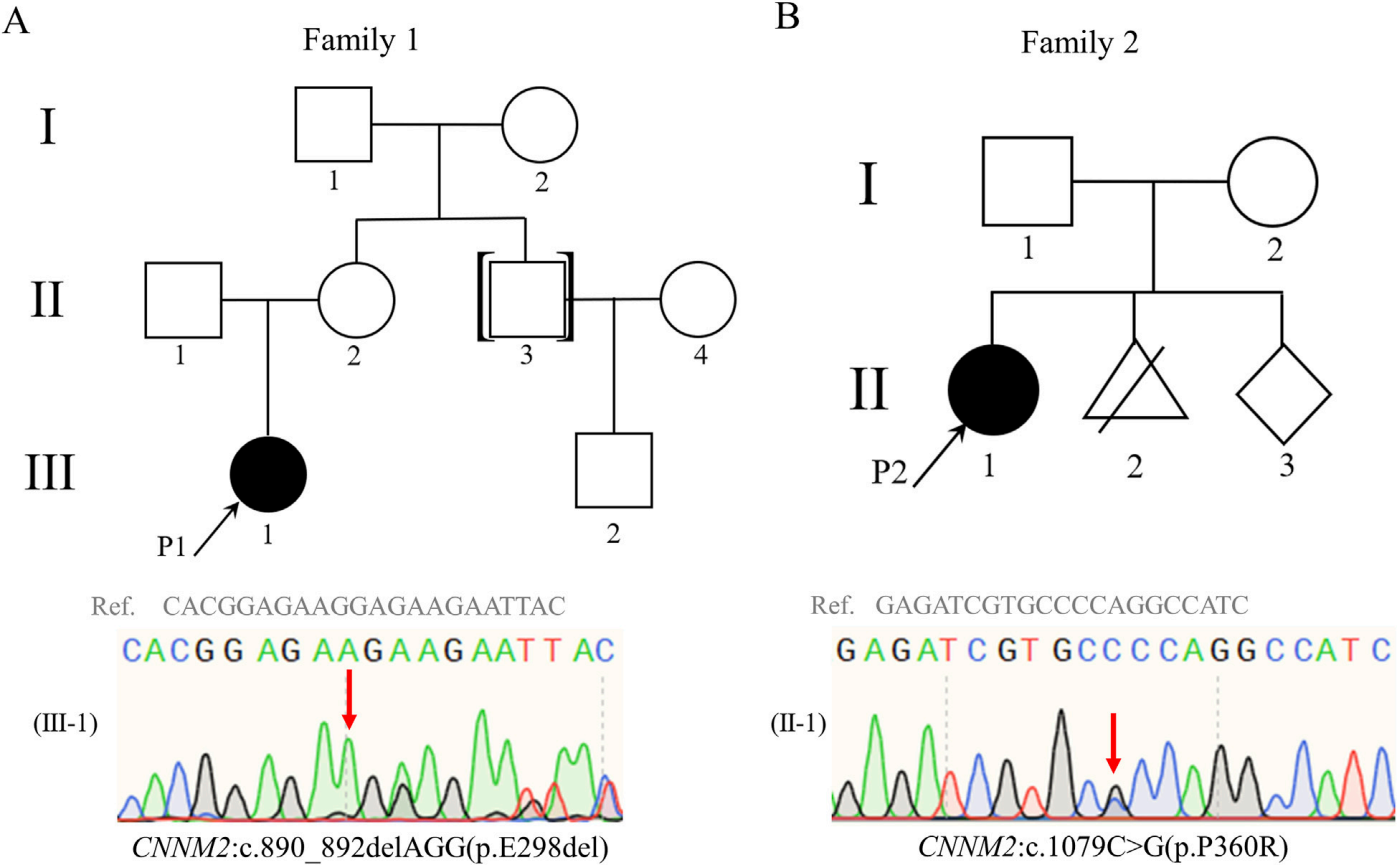
Pedigrees of family 1 and family 2. Both probands 1 **(A)** and 2 **(B)** carried heterozygous variants in *CNNM2* gene, and the variants were validated by Sanger sequencing. Red arrows indicate the locations of the variants; black arrows indicate the proband; black symbols indicate patients; white symbols indicate unaffected individuals. Abbreviations: Ref., reference sequence.

We employed PCR to amplify viable DNA from two family main members, followed by Sanger sequencing to validate candidate variants (Figure 1A,B; Supplementary Table S1). Sequence specific primers (Supplementary Table S1) were designed using Primer 5 software and produced by Sangon Biotech Co. Ltd. (Shanghai, China). SnapGene Viewer 6.0.2 (GSL Biotech, United States) and Seqman 7.1.0 (DNASTAR, Inc., Madison, WI) were used for data analysis.

### 2.4 Plasmid construction and site-directed mutagenesis

We performed functional analysis on two novel *CNNM2* variants of uncertain significance (c.890_892delAGG and c.1079C>G). Human WT *CNNM2* was amplified using normal human cDNA as a template, along with the corresponding primers: *CNNM2*-BamHI-F: 5′-CTT​GGT​ACC​GAG​CTC​GGA​TCC​GCC​ACC​ATG​ATT​GGC​TGT​GGC​GCT​TG-3′, and *CNNM2*-XhoI-R: 5′-GAA​GGG​CCC​TCT​AGA​CTC​GAG​GAT​GGC​GCC​TTC​GTT​GTG​CA-3’. Subsequently, the amplification products were subjected to double digestion and ligation into the pcDNA3.1-3xFlag-C vector, resulting in the generation of a WT cDNA plasmid. Two variant plasmids were obtained using the WT cDNA plasmid as a template, employing site-directed mutagenesis technique with the Mut Express II Fast Mutagenesis kit V2 (Vazyme, Nanjing, China). Primers used for site-directed mutagenesis were as follows: *CNNM2*-890_892-F: 5′-GAG​AAG​AAG​AAT​TAC​GCC​AAG​CGC​A-3′ and *CNNM2*-890_892-R: 5′-TTG​GCG​TAA​TTC​TTC​TTC​TCC​GTG​CCG​CAG​TTC​T-3′, *CNNM2*-1079-F: 5′-AGA​TCG​TGC​gCC​AGG​CCA​TCT​GCT​CCC​GG-3′ and *CNNM2*-1079-R: 5′-GAT​GGC​CTG​GcG​CAC​GAT​CTC​TCC​GAA​GAT​G-3’. All plasmids were verified by Sanger sequencing and extracted using the Endo-Free Plasmid DNA Maxi Kit (Omega Bio-Tek Inc., Guangzhou, China).

### 2.5 Cell culture and transfection

No *CNNM2* variants were detected in the HEK293T cells used for the *in vitro* analyses. HEK293T cells were cultured in DMEM (Gibco) supplemented with 10% fetal bovine serum (Gibco) and 1% penicillin–streptomycin (Gibco) at 37°C in a humidified 5% CO_2_ atmosphere. Cells were transfected with Lipofectamine 3,000 (Invitrogen) following the manufacturer’s instructions, with transfection efficiency exceeding 80% for each group.

### 2.6 RNA extraction and quantitative real-time PCR (qRT-PCR)

HEK293T cells (approximately 1 × 10^6^ cells per well) were seeded in 6-well plates. When the cells reached 70% confluence, they were transfected with 2.5 μg plasmid DNA using the Lipofectamine 3,000. 48 h (h) after transfection, total RNA from each group of cells was extracted using 1 mL TRIzol reagent (Invitrogen, Carlsbad, CA, United States) following the manufacturer’s instruction. The concentration and purity of RNA were determined using NanoDrop 1,000 (Thermo Fisher Scientific, MA, United States). The total RNA concentration for each group was greater than 500 ng/μL, with a purity (A260/A280 ratio) greater than 1.8. The cDNA was acquired using the RevertAid RT (Thermo Fisher, Carlsbad, CA, United States) as the template for PCR amplification. The qRT-PCR was performed on gene-specific primers for *CNNM2* and *GAPDH* (as an internal control) with Maxima SYBR Green qPCR Master Mix (Thermo Fisher, Carlsbad, CA, United States). The primer sequences were as follows: *CNNM2*-qPCR-F: 5′-TGA​AGC​TGG​GAA​AGA​AGG​TAT-3′ and *CNNM2*-qPCR-R: 5′-ACG​AGA​CAG​GGA​CAA​AGG​AA-3′, GAPDH-qPCR-F: 5′-GTG​GAC​CTG​ACC​TGC​CGT​CTA​G-3′ and GAPDH-qPCR-R: 5′-GAG​TGG​GTG​TCG​CTG​TTG​AAG​TC-3’. The relative changes of *CNNM2* expression were calculated with the comparative Ct (2^−ddCt^) method.

### 2.7 Protein extraction and Western blotting

HEK293T cells (approximately 1 × 10^6^ cells per well) were seeded in 6-well plates. When the cells reached 70% confluence, they were transfected with 2.5 μg plasmid DNA using the Lipofectamine 3,000. 48 h after transfection or treatment with cycloheximide (CHX, 50 μg/mL), HEK293T cells were lysed in SDS lysis buffer (Cat# P0013G, Beyotime, Jiangsu, China) containing protease inhibitor cocktail (1% volume of phenylmethanesulfonyl fluoride and P8340) according to standard procedures. The protein concentration was determined using the BCA protein assay kit (Cat# 23227, Thermo Fisher, Carlsbad, CA, United States). Then, the protein samples were separated by SDS-PAGE (approximately 25 μg per lane for *CNNM2* gene expression analysis and approximately 15 μg per lane for CHX-treated groups) and transferred onto a polyvinylidene fluoride (PVDF) membrane (Merck Millipore, Burlington, MA, United States). The PVDF membranes were incubated with a blocking solution consisting of 5% (w/v) skim milk powder in PBS containing 0.1% Tween and protein expression was detected by primary and secondary antibodies. CNNM2 protein was collected using a 1:4,000 dilution of mouse DYKDDDDK tag monoclonal antibody (Cat# 66008-4-Ig, Proteintech, Chicago, United States), and the internal reference was diluted with mouse anti-GAPDH antibody (Cat# 200306-7E4, ZENBIO, Chengdu, China) at 1:2000 and rabbit β-tubulin polyclonal antibody (Cat# GTX101279, GeneTex, Irvine, United States) at 1:1,000. Grayscale analysis was performed using ImageJ software.

### 2.8 Confocal microscopy

HEK293T cells (approximately 2 × 10^5^ cells per well) were seeded on glass coverslips in 24-well plates overnight, and they were transfected with 0.5 μg plasmid DNA using the Lipofectamine 3,000. After 48 h, then fixed in 4% paraformaldehyde. Following permeabilizing with 0.3% Triton X-100 for 15 min, the cells were blocked in 5% bovine serum albumin (BSA) for 1 h. Then, cells were incubated with mouse DYKDDDDK tag monoclonal antibody (1:1,000) and rabbit β-catenin polyclonal antibody (1:200, Cat# 51067-2-AP, Proteintech, Chicago, United States) in 5% BSA at 4 °C overnight. The following day, the cells were washed with PBS and incubated with fluorescent-labeled secondary antibody (Cy™3 AffiniPure Goat Anti-Mouse IgG and Alexa Fluor 488 AffiniPure Goat Anti-Rabbit IgG) at a dilution of 1:200 in 5% BSA for 1 h at room temperature in the dark. Nuclei were stained with 4′,6-diamidino-2-phenylindole (DAPI) (Cat# C1002, Beyotime, Jiangsu, China) for 5 min. Coverslips were shielded using Fluoromount™ Aqueous Mounting Medium (Sigma Aldrich, St. Louis, MO, United States). Finally, the cells were imaged using a TCS SP5 laser confocal microscopy (Leica, Wetzlar, Germany).

### 2.9 Flow cytometry analysis

HEK293T cells (approximately 1 × 10^6^ cells per well) were seeded in 6-well plates. When the cells reached 70% confluence, they were transfected with 2.5 μg plasmid DNA using the Lipofectamine 3,000. 48 h after transfection, intracellular magnesium levels in HEK293T cells were assessed using Mag-Fluo-4 a.m. (Cat# MX4544, Maokang Biotech, Shanghai, China) as directed by the manufacturer. The cells in each group were incubated at 37°C for 10 min with Hanks’ Balanced Salt Solution containing Mg^2+^ (Cat# C0219, Beyotime, Jiangsu, China). Then, 4 μL of 2.5 mM Mag-Fluo-4 a.m. stock solution and 5 μL of 20% Pluronic^®^ F-127 (Cat# MS4302, Maokang Biotech, Shanghai, China) were added to 1 mL of complete culture medium and vigorously vortexed to prepare the probe dispersion. Subsequently, 250 μL of the probe dispersion was added to 750 μL of cell-containing medium, mixed thoroughly to achieve a final concentration of 2.5 μM working solution, and incubated at 37°C for 30 min. After incubation, the cells were washed three times with PBS and subsequently incubated in Mg^2+^-free Hanks’ Balanced Salt Solution (Cat# C0218, Beyotime, Jiangsu, China) at 37°C for an additional 30 min to ensure Mg^2+^ depletion and complete de-esterification of the intracellular AM esters. Fluorescence intensities were measured in triplicate assays using flow cytometry (Cytek, United States) at an excitation wavelength of 488 nm and an emission wavelength of 525 nm. Data analysis was conducted using FlowJo software (version 10.8.1, BD Biosciences).

### 2.10 Statistical analysis

All experiments were performed three times, and the data were presented as mean ± standard deviation (SD). Statistical analysis was conducted using GraphPad Prism 9.0 software. Normality test and Student’s t-test (two-tailed) were used to compare differences between two groups, and *p* < 0.05 was considered statistically significant.

## 3 Results

### 3.1 Clinical features

Proband 1, a female, was the firstborn child of non-consanguineous parents from their third pregnancy, delivered at preterm (34^+^ weeks) via caesarean section, without a history of perinatal asphyxia or hypoxia. Her mother had no significant medical history during pregnancy. She started lifting her head and rolling over at the same age as her peers. At 7 months of age, caregivers noticed that she exhibited poorer responses compared to peers and showed less interest in toys. At 9 months of age, she experienced her first epileptic seizure and sought medical attention. The electroencephalogram (EEG) demonstrated sleep stage-specific waveforms, with occasional suspicious sharp waves appearing synchronously or asynchronously in bilateral frontal and central regions. Brain Magnetic Resonance Imaging (MRI) revealed symmetric bilateral abnormal signals in the posterior periventricular white matter, suggesting the possibility of delayed terminal myelination, accompanied by slight widening of the bilateral frontal and temporal subarachnoid spaces. These signals appeared slightly hyperintense on T1-weighted images (T1WI), slightly prolonged signal on T2-weighted images (T2WI), slightly high signal on fluid-attenuated inversion recovery (FLAIR). She received relevant symptomatic treatment, but the outcome was not satisfactory.

She presented with distinctive facial features, characterized by a wide interocular distance, flat nasal bridge, short nasal tip, and bilateral lateral canthal slanting. She began vocalizing “mama” and “baba” at 1 year and 3 months of age and started walking at 1 year and 6 months. Currently, at 5 years of age, she measured 120 cm in height, weighed 25 kg, and had a head circumference of 54 cm. Despite this, she lacked liveliness, exhibited slightly delayed responses to external stimuli, and in terms of motor skills, she could run but had poor jumping ability and difficulty navigating stairs. There is no similar medical history within this family.

Proband 2, a female, was the firstborn of the non-consanguineous parents, delivered at full term via spontaneous vaginal delivery, without a history of perinatal asphyxia or hypoxia. Apgar score indicated no abnormalities. Her mother had no significant medical history during pregnancy. At birth, she weighed 3.650 kg and measured 51 cm in length, but the head circumference was not provided. She was breastfed until 1 year and 2 months of age, and then began crawling at the age of 10 months, achieved independent standing at 1 year and 6 months, started walking at 1 year and 8 months, and vocalized “mama” and “baba” at the age of 2 years.

At the age of 3 years and 11 months, she presented for the first time due to delayed speech development (limited to a few single words) and abnormal gait. She underwent assessment using the Reynell Developmental Language Scales III (RDLS-III), scoring 18 points, which is equivalent to 2.01 years of age. Both her fine motor development quotient (FMQ) and gross motor development quotient (GMQ) were below 1%. Brain MRI did not show any obvious abnormalities. Following rehabilitation therapy including language, cognition, and sensory integration, her symptoms did not show significant improvement. Currently, she was 5 years old, with a height of 120 cm, a weight of 25 kg, and a head circumference of 48.6 cm. Otherwise, she was unfocused. She has not yet experienced seizures, and the EEG showed no substantial abnormalities. There is no similar medical history within her family.

The clinical phenotypes of the two probands are listed in Table 1. To further clarify the etiology and potentially conceive another healthy child, each of them visited our medical genetics outpatient clinic for evaluation. We suspected that they might be suffering from neurodevelopmental disorders and conducted relevant genetic tests.

**TABLE 1:** Clinical features of proband 1 and proband 2.

Patient ID	Proband 1 (family1, III-1)	Proband 2 (family2, II-1)
Gestation	Preterm birth (34^+^ weeks)	Full term
Gender, age at the last exam	Female, 5 years	Female, 5 years
Height	120 cm (+2SD∼+3SD)	112 cm (0SD∼+1SD)
Weight	25 kg (+2SD∼+3SD)	23 kg (+1SD∼+2SD)
BMI	17.36 kg/m^2^ BMI percentile ≥85 and <95	18.34 kg/m^2^ BMI percentile ≥95
Head circumference	54 cm (+2SD∼+3SD)	48.6 cm (-1SD∼0SD)
Intellectual disability	Mild	Severe
Seizures	+	-
Developmental delay	+	+
Dyskinesia	+	+
Delayed speech	-	+
Abnormal mental behavior	Autism spectrum-like and aggressive behaviors	Inattentive
Craniofacial features	Wide interocular distance, flat nasal bridge, short nasal tip, bilateral lateral canthal slanting	-
Hypertension	-	-
Sleep apnea	-	-
Myocardial infarction	-	-
Other phenotypes	-	-
Serum magnesium level (0.74–1.07 mmol/L)	0.62 mmol/L	0.56 mmol/L
24 h Urinary magnesium level (3-4.5 mmol/24 h)	NA	3.14 mmol/24 h
EEG	Suspected sharp waves in bilateral frontal and central regions during sleep	No abnormality
Brain MRI	Bilateral symmetric abnormal signals in the posterior periventricular white matter, suggesting the possibility of delayed terminal myelination, accompanied by slight widening of the bilateral frontal and temporal subarachnoid spaces	No abnormality
Family history	-	-

Abbreviations: BMI, body mass index; BMI, percentile≥95: obesity, BMI, percentile≥85 and<95: overweight (According to the World Health Organization guidelines, 2019, and BMI, after correction for age); EEG, electroencephalogram; MRI, magnetic resonance imaging; SD, standard deviation; NA, not applicable; (+), presence; (-), absence.

### 3.2 Genetic tests

The G-banded chromosomal karyotype of proband 1 and proband 2 was 46, XX, with no apparent abnormalities detected in their biological parents. No chromosomal aneuploidy or copy number variations larger than 100 kb, known to be pathogenic, were detected in copy number variation sequence (CNV-seq) of proband 1 and proband 2. Trio-whole exome sequencing (trio-WES) analysis identified a candidate heterozygous variant in proband 1, *CNNM2*(NM_017649.5): c.890_892delAGG, and a separate candidate heterozygous variant in proband 2, *CNNM2*(NM_017649.5): c.1079C>G (Figures 1A,B). Meanwhile, their parents were wild type (WT) at the corresponding loci (Supplementary Figure S1). Two candidate variants were confirmed through Sanger sequencing (Supplementary Figure S1; Supplementary Table S1). Both variants were associated with the clinical phenotype of the probands, but their pathogenic significance were uncertain according to American College of Medical Genetics and Genomics (ACMG) standards (Richards et al., 2015). To further confirm the pathogenicity of these two candidate variants, we initiated relevant functional studies.

### 3.3 In *silico* analysis

**FIGURE 2:**
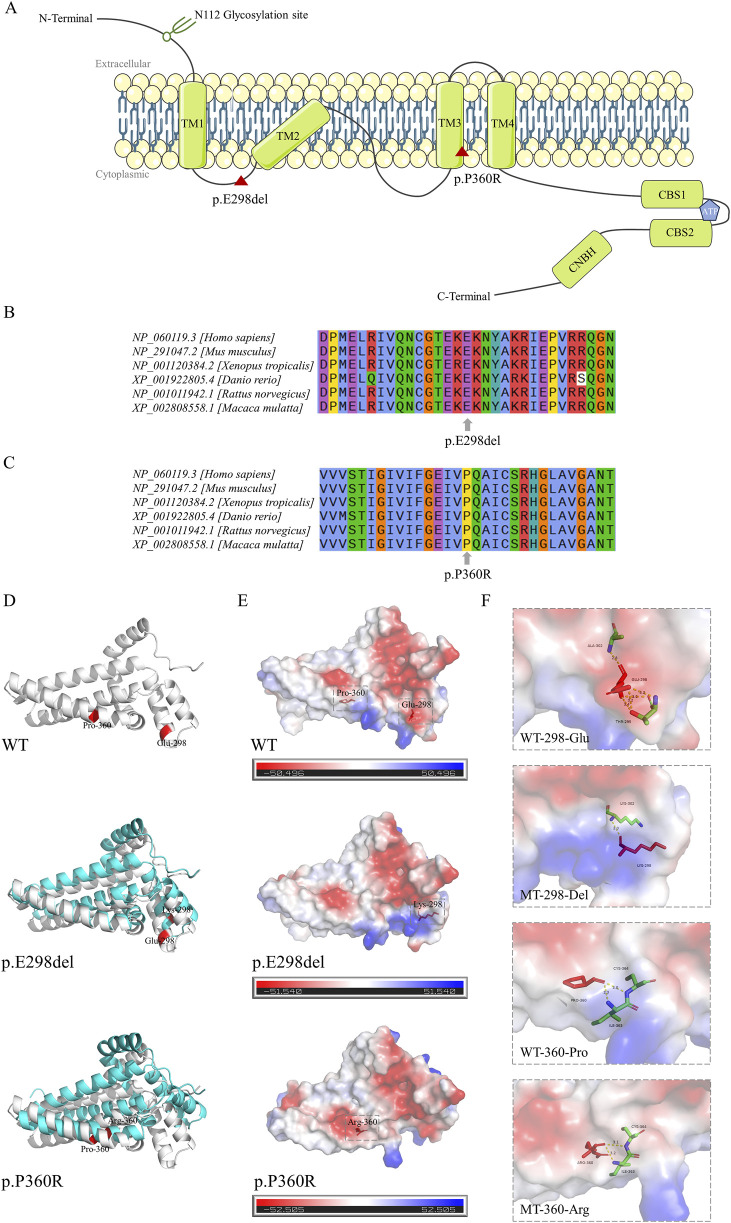
Location diagram and *silico* analysis of *CNNM2* variants. **(A)** The diagram of CNNM2 protein domains. Red triangle symbol indicates the location of p.E298del and p.P360R variants. **(B,C)** Conservation analysis of amino acids E298 and P360 across multiple species. **(D)** Three-dimensional structural models of the CNNM2 protein transmembrane domains. The wild type (WT) structure was represented in gray, the mutant type (MT) structures in blue, and the positions of the variant amino acids are highlighted in red. **(E)** Surface charge distribution of the CNNM2 protein transmembrane domains. Blue represented positive charge, red represented negative charge, white represented neutral charge, and the red stick model indicated the position of the variant amino acids. **(F)** Schematic of the hydrogen bonding interactions surrounding the variant sites of *CNNM2* WT, p.E298del, and p.P360R. The variant amino acid positions were represented by red stick models, surrounding amino acids by colored stick models, and hydrogen bonds between amino acids were depicted by yellow dashed lines. The hydrogen bond size was indicated by numerical values.

Both the p.E298del and p.P360R variants are located in the transmembrane structural domain (Figure 2A). Conservation analysis using T-Coffee software indicated that the locations of the two variants were highly conserved across a broad range of species (Figures 2B,C). These two variants were absent in the general population and were predicted to be damaging by several bioinformatics tools (Table 2). Specifically, the p.E298del variant was predicted to be disease-causing (score = 1) by MutationTaster. The p.P360R variant was predicted to be disease-causing (score = 1) by MutationTaster, probably damaging (score = 1) by PolyPhen-2, damaging (score = 0) by SIFT, and damaging (score = 0.992) by REVEL. These predictions strongly suggest that both variants may impair the structure and function of the CNNM2 protein.

**TABLE 2:** Two novel *CNNM2* variants are predicted to be damaging.

Probands	Nucleotide change	Source	Inheritance	MAF (ExAC or 1000G)	MutationTaster	Polyphen-2	SIFT	REVEL	ACMG Classification	Novel
P1	c.890_892delAGG, p.E298del	Het, *de novo*	AD	0	Disease_causing	NA	NA	NA	LP, (PS2_Supporting, PS3_Moderate PM2_Supporting PM4, PP3)	Y
P2	c.1079C>G, p.P360R	Het, *de* *novo*	AD	0	Disease_causing	1.0	0	0.992	LP, (PS2_Supporting, PS3_Moderate PM2_Supporting PP2, PP3)	Y

Abbreviations: MAF, minimum allele frequency; ACMG, american college of medical genetics and genomics; Het, heterozygous; NA, not applicable; LP, likely pathogenic; Y, yes.

In the computationally predicted three-dimensional structural models of the CNNM2 protein transmembrane domains, the p.E298del and p.P360R variants caused varying degrees of structural displacement (Figure 2D). The p.E298del variant resulted in the substitution of Glu298 with Lys298, significantly altering its hydrogen bonding interactions and sizes with surrounding amino acids. This variant deleted the hydrogen bonds between Lys298 and Thr295, as well as Ala302, while forming a new hydrogen bond with Lys302, leading to a shift in local surface charge from negative to positive (Figures 2E,F). In the p.P360R variant model, the hydrogen bond size between Arg360 and Ile363 was reduced, whereas the hydrogen bond size between Arg360 and Cys364 was increased, resulting in a change in local surface charge from positive to neutral (Figures 2E,F).

### 3.4 Variants increased *CNNM2* gene expression levels and impaired the cell membrane localization of the CNNM2 protein

**FIGURE 3:**
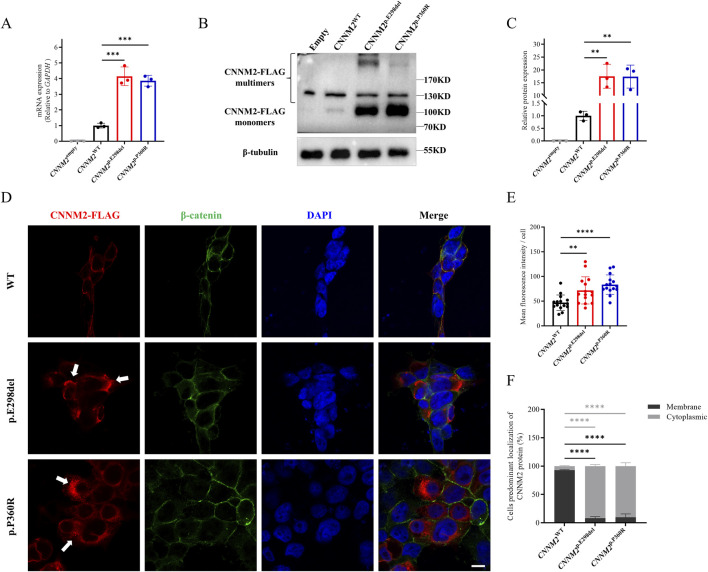
Relative transcription and translation analysis of the *CNNM2* variants *in vitro* (HEK293T cells after transfection with mutant plasmids). **(A)** Quantitative PCR showed that the mRNA expression of the p.E298del and p.P360R variants were higher than that of WT. The data were based on three independent biological replicates. **(B,C)** Western blotting analysis, along with quantification, revealed that the protein expression of the p.E298del and p.P360R variants were elevated compared to the WT. The data were based on three independent biological replicates. **(D)** Cells were immunostained with FLAG antibodies (red), β-catenin antibodies (green) and nuclei were counterstained with DAPI (blue). White arrows indicated the aggregation of FLAG-tagged CNNM2 protein in the cytoplasm. Scale bar, 10um. **(E)** Quantitative analysis of mean fluorescence intensity of each transfected cell. The data were obtained from three independent biological replicates, with five oil-immersion fields (630X) of view analyzed per replicate, each field containing more than 20 transfected cells. **(F)** Quantify the percentage of cells with predominant membrane vs cytoplasmic CNNM2 signal. The data were based on three independent biological replicates, with at least 50 cells analyzed per condition. (t-test, ns: no significance; **: P < 0.01; ***: P < 0.001; ****: P < 0.0001).

To verify whether the two variants caused any functional changes in *CNNM2*, we constructed eukaryotic overexpression vectors carrying the mutants or WT *CNNM2*, and transfected them into HEK293T cells. The mRNA expression levels of the WT, p.E298del and p.P360R were higher than those of the empty control. The quantitative real-time PCR (qRT-PCR) results showed that, compared with the WT, the p.E298del and p.P360R variants led to an increase in the transcription levels (Figure 3A). Meanwhile, there was no difference in GAPDH expression between all groups. Additionally, the protein levels of the two variants were also shown to be increased, as detected by Western blotting (Figures 3B,C).

Next, we employed immunofluorescence to observe the subcellular localization of CNNM2 protein. The results showed that WT CNNM2 was primarily localized near the cell membrane (Figure 3D). Interestingly, we found that the expression of CNNM2 protein induced by p.E298del and p.P360R variants was significantly higher than that of the WT, which was consistent with the Western blotting results (Figure 3E). These abnormally elevated variant proteins mainly aggregated in the cytoplasm, forming clumps (Figures 3D,F).

### 3.5 Variants led to intracellular Mg^2+^ retention

**FIGURE 4:**
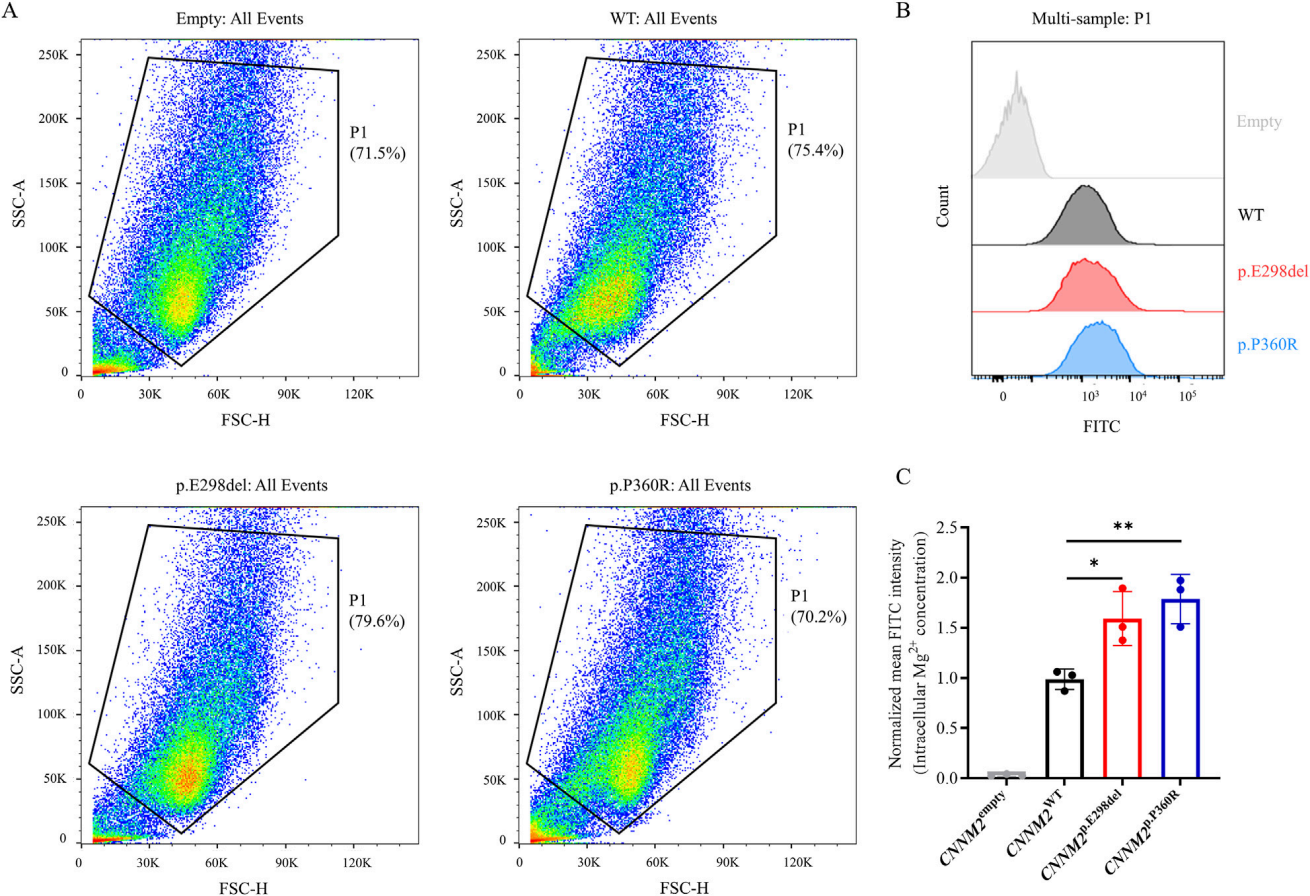
Intracellular Mg^2+^ of transiently transfected HEK293T cells with FLAG-tagged CNNM2 variants using flow cytometry. **(A,B)** Using the Mag-Fluo-4-AM indicator to label intracellular Mg^2+^, p.E298del, and p.P360R variants increased intracellular Mg^2+^ levels compared to WT. **(C)** Quantitative analysis of intracellular Mg^2+^ levels. (t-test, *: P < 0.05; **: P < 0.01). The data were based on three independent biological replicates.

CNNM2 is one of the important members of the Mg^2+^ transport family, primarily responsible for mediating Mg^2+^ efflux. The p.E298del and p.P360R variants impaired membrane localization of the CNNM2 protein, but it remains unclear whether this affects Mg^2+^ transport function. Subsequently, we used the Mag-Fluo-4-AM indicator to label intracellular Mg^2+^ in transfected cells and detected it using flow cytometry. The results showed that p.E298del and p.P360R variants increased intracellular Mg^2+^ levels compared to WT (Figures 4A–C). This suggested that p.E298del, and p.P360R variants may affect Mg^2+^ efflux mediated by CNNM2.

### 3.6 Variants decreased CNNM2 protein stability

**FIGURE 5:**
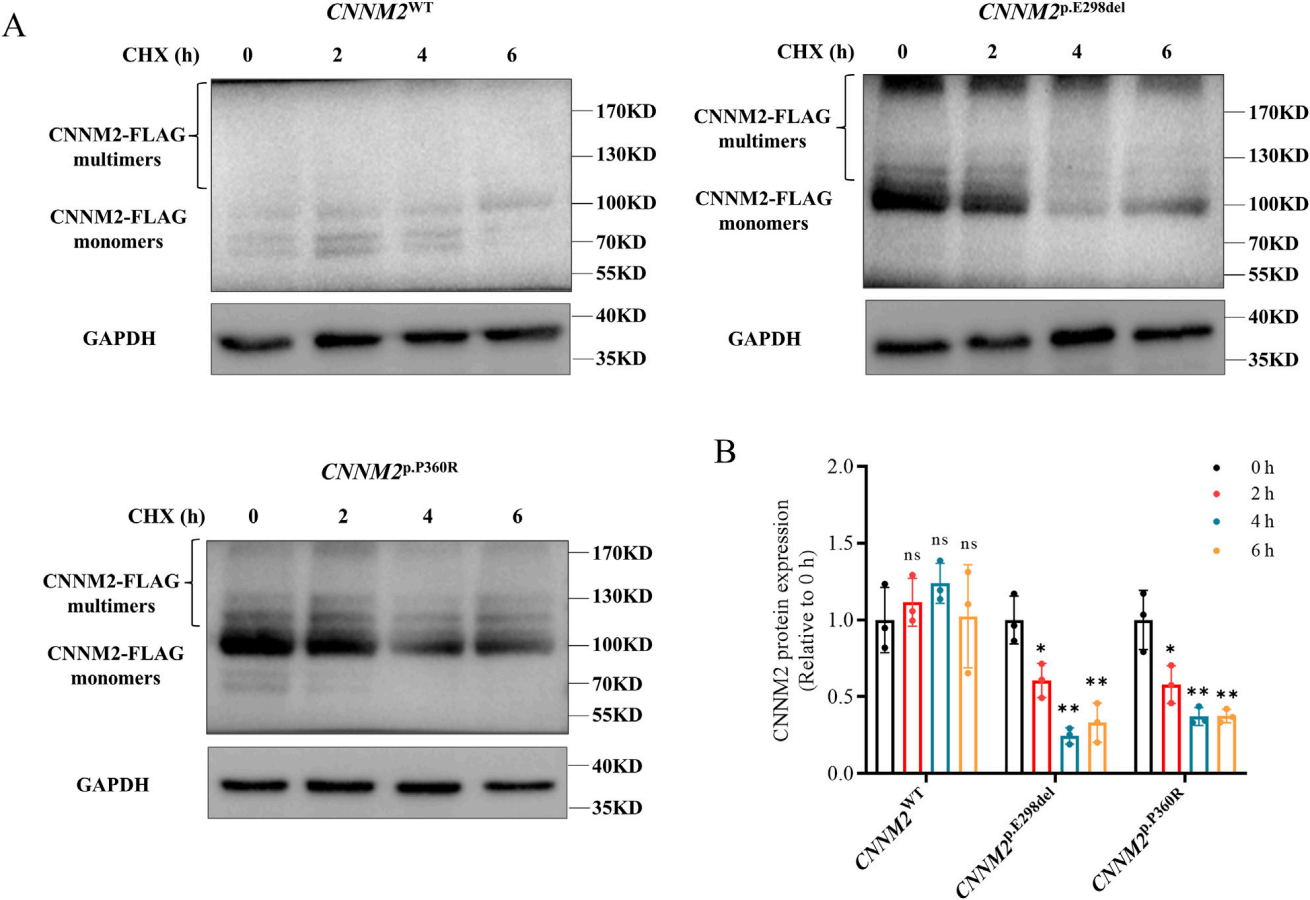
The stability of CNNM2 variant proteins in transiently transfected HEK293T cells. **(A,B)** Western blotting analysis and quantification showed that the p.E298del and p.P360R variant proteins degraded earlier than WT after CHX treatment. (t-test, ns: no significance; *: P < 0.05; **: P < 0.01). The data were based on three independent biological replicates.

The p.E298del and p.P360R variants resulted in increased expression of the *CNNM2* gene, but with impaired Mg^2+^ transport function. We further explored the potential pathogenic mechanisms. CHX is a commonly used inhibitor of protein synthesis, typically employed in cell culture to block the production of newly synthesized proteins. To assess the stability of CNNM2 protein, we transfected HEK293T cells and treated them with CHX for 0, 2, 4, and 6 h (h). The expression of CNNM2-FLAG protein was evaluated by Western blotting. In the control group, the protein levels of CNNM2-FLAG in WT cells did not exhibit a significant decrease over time with CHX treatment (Figures 5A,B). However, the levels of p.E298del and p.P360R variant proteins showed a notable decrease after 2 h of CHX treatment (Figures 5A,B). This suggested that p.E298del and p.P360R variants may affected the stability of CNNM2 protein, accelerating its degradation.

## 4 Discussion

Currently, 30 variants involving 41 patients have been associated with *CNNM2* related NDDs (Supplementary Table S2) (Arjona et al., 2014; Accogli et al., 2019; Bamhraz et al., 2021; Franken et al., 2021a; Huang et al., 2021; Li et al., 2021; Panda et al., 2021; Zhang et al., 2021; Petrakis et al., 2022; Tseng et al., 2022; Xu et al., 2022; Liu C. X. et al., 2023; Wang et al., 2023; Bosman et al., 2024). HOMGSMR1 showed significant clinical heterogeneity, with different variant sites presenting distinct phenotypes and inheritance patterns. However, the understanding of the genotype-phenotype relationship remains insufficient. Many variants of uncertain significance severely compromise the effectiveness of genetic counseling. In our study, we identified two novel pathogenic *CNNM2* variants through genetic testing and functional studies, p.E298del and p.P360R, which were initially classified as VUS according to the ACMG criteria. Subsequently, genetic counseling and prenatal diagnosis were performed in family 2, resulting in the identification of an unaffected fetus.

Both probands exhibited intellectual disability, developmental delay, hypomagnesemia, overweight, and abnormal behaviors, which overlap with the main phenotype of HOMGSMR1. Notably, proband 1 also presented with facial dysmorphism and mild brain imaging abnormalities, while proband 2 has not yet displayed signs of seizures. Among the reported cases, facial dysmorphism was only mentioned in homozygous p.V548M (Accogli et al., 2019) variant proband, with features such as enlarged nares, thick and uplifted earlobes, which differ from our proband 1. Therefore, the unique facial features of proband 1 may serve to expand the phenotype of HOMGSMR1. Additionally, brain MRI in proband 1 revealed delayed terminal myelination, but unfortunately, we were unable to obtain the original images. In previous cases, brain MRI abnormalities were observed in only two homozygous variant patients (p.E122K (Arjona et al., 2014), p.V548M (Accogli et al., 2019)) and a small number of heterozygous variant patients (p.P482A (Petrakis et al., 2022), p.V483GfsTer29 (Wang et al., 2023), p.R797X (Franken et al., 2021a)), which included cerebral cortical atrophy, myelination defects, local enlargement of the subarachnoid space, and isolated demyelinating-type lesions near the corpus callosum. The brain imaging features of these variants were similar. We identified a novel heterozygous variant, p.E298del, and further confirmed the importance of *CNNM2* in brain structural development, thereby supporting the genotype-phenotype relationship. Therefore, when encountering such patients, prompt attention should be given to changes in brain imaging to facilitate more accurate diagnosis.

Hypomagnesemia is one of the main phenotypes of HOMGSMR1, and its late diagnosis may lead to more severe neurodevelopmental outcomes (Accogli et al., 2019). Therefore, based on the results of genetic testing, we promptly conducted serum Mg^2+^ level testing for both patients, which indicated that their serum magnesium levels were below the normal range. They denied taking any medications known to cause low Mg^2+^ levels. Magnesium is crucial for the maintenance of neuronal growth and development, myelination, synaptogenesis, and signal transmission (Xu et al., 2014; Yamanaka et al., 2019). Low serum Mg^2+^ concentrations are associated with various neurological disorders, including intellectual disability and seizures (Andrási et al., 2000; Hermosura et al., 2005; Mele et al., 2021). Although HOMGSMR1 patients exhibited refractory hypomagnesemia, with serum Mg^2+^ levels remaining below the normal range despite oral or intravenous magnesium supplementation, some patients showed a reduction in the frequency of seizures (Tseng et al., 2022; Xu et al., 2022; Liu C. X. et al., 2023). However, the guardian of our patients temporarily refused magnesium supplementation. Currently, the primary approach is dietary management, focusing on the consumption of magnesium-rich foods such as grains, cereals, and dark leafy vegetables. Interestingly, proband 2 has not experienced any seizures to date. Most of the probands reported exhibited varying degrees of seizures, but probands with the p.A92P (Bosman et al., 2024), p.Y314X (Franken et al., 2021a), p.G339D (Franken et al., 2021a), p.M383V (Bosman et al., 2024), p.G437E (Bosman et al., 2024), p.P482A (Petrakis et al., 2022), and p.S795L (Franken et al., 2021a) variants did not display seizures. These variants are spread across the entire *CNNM2* gene without any clustering, and the mechanisms underlying the absence of seizures could require further investigation. Additionally, disturbances in magnesium homeostasis in HOMGSMR1 patients may be associated with other electrolyte imbalances, such as hyperparathyroidism, hypocalciuria, and hypocalcemia detected in patients with p.V548M (Accogli et al., 2019) and p.Y189C (Xu et al., 2022) variants, as well as hypercalcemia observed in *Cnnm2* ^*−/+*^ mice. However, this association remains controversial, and no calcium ion disturbances were detected in our two probands.

Our patients exhibited an increase in body mass index (BMI), and the association between *CNNM2* and BMI has been established. The SNP rs12411886 has been reported to be associated with high BMI (Lv et al., 2017), while the G allele of rs12413409 has been linked to a reduced body mass index (Nakagami, 2015; Franken et al., 2021a) suggested that obesity may be a major feature of HOMGSMR1 syndrome, occurring in approximately 89% of cases. However, such a feature was not observed or mentioned in some sporadic family probands. Interestingly, among two unrelated probands, both carrying the heterozygous p.E357K variant, one had severe obesity, while the other did not present with an obesity phenotype (Arjona et al., 2014). As more probands are identified, the proportion of obesity seems to be decreasing. This suggests that future studies on HOMGSMR1 could focus on monitoring the BMI of patients to provide further evidence for this phenotypic feature. In addition, *CNNM2* has also been implicated in the development of hypertension (Funato et al., 2017), intracranial aneurysm (Liu M. et al., 2023; Wu et al., 2024), myocardial infarction (Matsuoka et al., 2015), pulmonary hypertension (Wang et al., 2021), and sleep apnea (Gui et al., 2024). However, no such abnormalities have been observed in our patients thus far. This will be one of the key points for further monitoring during subsequent follow-ups, and may also be applicable to other HOMGSMR1 patients.

It is noteworthy that the p.E298del and p.P360R variants of *CNNM2* described in our study are the first reported at these specific positions, and both are located within the transmembrane domains. By combining our findings with existing reports, a total of 32 variant sites associated with NDDs have been identified. Among these 32 variants, the majority are heterozygous, with only 2 being homozygous (p.E122K (Arjona et al., 2014), p.V548M (Accogli et al., 2019)). The majority of the variants are missense mutations (24/32), followed by nonsense mutations (2/32), exon deletions (2/32), in-frame deletions (3/32), and frameshift mutations (1/32). Notably, 10 of the variants are clustered in the 321–366 residue region within the transmembrane domains. This suggests that the 321–366 residue region may be a high-frequency or hotspot variant area for the *CNNM2* gene, although this hypothesis requires further validation and exploration through additional clinical cases.

Initially, both the p.E298del and p.P360R variants were classified as VUS according to the ACMG criteria. Nevertheless, through functional experiments, we confirmed the pathogenicity of p.E298del and p.P360R variants and observed that they led to an increase in *CNNM2* transcription and total protein levels. The increased CNNM2 protein accumulated in the cytoplasm, a phenomenon that was consistent with observations at other known variant sites, including p.A92P (Bosman et al., 2024), p.E122K (Arjona et al., 2014), p.S269W (Arjona et al., 2014), among others. However, for variants such as p.L48P (Franken et al., 2021a), p.L330F (Arjona et al., 2014), p.E357K (Arjona et al., 2014), p.R480L (Tseng et al., 2022), and p.R480K (Tseng et al., 2022), the increased protein did not affect subcellular localization but rather aggregated at the cell membrane, and the molecular mechanism underlying this phenomenon remains unclear. Previous studies have shown that the N-terminal extracellular domain of *CNNM2* plays a crucial role in the proper localization of the protein to the plasma membrane, with N-glycosylation at Asn-112 being particularly essential for the stability of CNNM2 on the membrane (de Baaij et al., 2012). Computational predictions indicated that p.E298del and p.P360R variants could lead to abnormal hydrogen bond connections around the mutation site, transmembrane domain abnormalities, and surface charge changes. However, it remains to be further validated whether these mutations affect plasma membrane localization by influencing N-glycosylation at Asn-112.

The intracellular Mg^2+^ concentration in HEK293T cells with the p.E298del and p.P360R variants was higher than that of the WT, suggesting that these two variants may cause a dysfunction in magnesium efflux, leading to magnesium homeostasis disruption. Further speculation suggests that the increased CNNM2 protein expression may result from a negative feedback mechanism due to impaired Mg^2+^ transport function in the mutants. The dysfunction in Mg^2+^ efflux may be the cause of hypomagnesemia in these two patients. However, whether CNNM2 directly or indirectly mediated Mg^2+^ transport remained controversial. Most studies suggested that CNNM2 itself was not a transporter protein. CNNM2 might have functioned as a cytoplasmic Mg^2+^ sensor or as an Mg^2+^-sensing mechanism, involved in regulating the magnesium ion transport activities of TRPM7 and SLC41A3 transporters, although the exact regulatory mechanisms were not fully understood (de Baaij, 2015; Li et al., 2017). But, a few studies proposed that the CBS domain and DUF21 region may directly interacted with Mg^2+^, thereby contributing to Mg^2+^ efflux (Hirata et al., 2014; Corral-Rodríguez et al., 2014; Huang et al., 2021). Overall, the molecular pathogenic mechanisms of CNNM2 remained unclear. Unlike previous studies, we may have discovered potentially new insights into the pathogenic mechanisms of CNNM2. We found that the protein stability of these two *CNNM2* variants was significantly reduced and prone to degradation. This may be one of the reasons why the variants lead to disruption in CNNM2 mediated Mg^2+^ transport. Future studies could reveal further pathogenic mechanisms through which CNNM2 contributes to neurodevelopmental disorders by utilizing additional cell models, such as SH-SY5Y cells or iPSC-derived neurons.

In brief, based on bioinformatics analysis and functional study findings for the two variants, they could be classified as “likely pathogenic” (LP), which provides a definitive diagnosis and precise treatment management for the proband 1 and 2. Currently, HOMGSMR1 is primarily treated with magnesium supplementation, mainly through oral and intravenous magnesium agents. Additionally (Petrakis et al., 2022), found that spironolactone, by inhibiting aldosterone, reduced magnesium loss in urine and alleviated hypomagnesemia in HOMGSMR1 patients, making it a promising magnesium-sparing agent. Moreover, Magnesium L-threonate, which has received GRAS (Generally Recognized As Safe) certification by the U.S. FDA (GRN No. 499), has been shown to effectively increase magnesium levels in the brain and neurons (Slutsky et al., 2010). In *Drosophila*, dietary magnesium supplementation has been shown to enhance long-term memory (Wu et al., 2020). This effect is mediated by the unextended (*UEX*) gene, which encodes a homolog of the mammalian Cyclin M2 Mg^2+^-efflux (Wu et al., 2020). Studies have shown that UEX-driven Mg^2+^ efflux is critical for the slow rhythmic regulation of Mg^2+^ levels in Kenyon cells, which are the principal neurons of the mushroom body involved in memory processing (Wu et al., 2020). Notably, mutant flies lacking UEX exhibit significant memory impairments. Moreover, Mg^2+^ is a positive regulator of synaptic plasticity. In cultured rat hippocampal neurons, increasing the extracellular Mg^2+^ concentration within the physiological range enhances synaptic plasticity, an effect closely associated with increased hippocampal synaptic density and upregulation of NR2B subunit expression of NMDA-type glutamate receptors (Slutsky et al., 2004; Slutsky et al., 2010). Furthermore, in cultured rat infralimbic prefrontal cortex neurons, elevating extracellular Mg^2+^ concentration also enhances synaptic NMDAR current and plasticity (Abumaria et al., 2011). Therefore, following a definitive diagnosis, blood magnesium levels may be elevated through various magnesium supplementation therapies, although most patients still have subnormal levels after treatment. We will continue to follow up with family 1 and family 2, and provide timely recommendations for magnesium supplementation based on individual circumstances. Meanwhile, we have also assisted family 2 in obtaining a healthy fetus through prenatal diagnosis.

## 5 Conclusion

In this study, we described two Chinese HOMGSMR1 families and identified two novel variants in *CNNM2* gene, c.890_892delAGG (p.E298del) and c.1079C>G (p.P360R), which were classified as likely pathogenic after functional validation. Our study expands the mutation and phenotypic spectrum, as well as the functional studies of *CNNM2*, and contributes to genetic testing and prenatal diagnosis in families with HOMGSMR1.

## Data Availability

The datasets presented in this study can be found in online repositories. The names of the repository/repositories and accession number(s) can be found below: https://ngdc.cncb.ac.cn/search/specific?db&equals;hra&q=HRA010873, HRA010873.
