# Supplementary material for: Two novel variants in CNNM2 disrupts magnesium efflux leading to neurodevelopmental disorders
Source: Front Genet. 2025 Jun 19;16:1600877. doi: 10.3389/fgene.2025.1600877 (PMC12222124; doi:10.3389/fgene.2025.1600877)
Supplement: Supplementary file 1 [file Supplementaryfile1.docx]

***Supplementary Material***

**Supplementary Figures and Tables**

**Supplementary Figures**


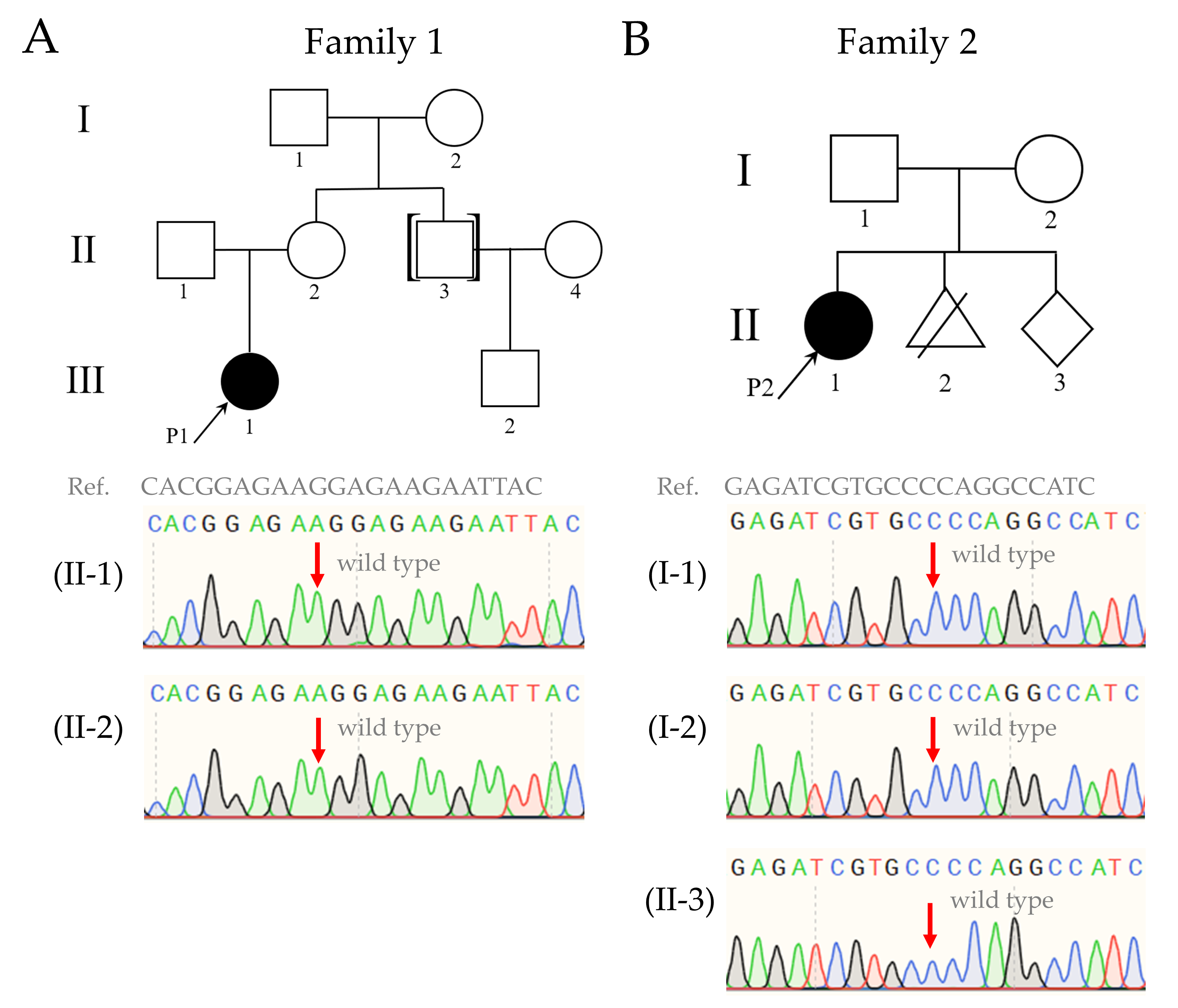


**Supplementary Figure 1.** Sanger sequencing of the main members from family 1 and family 2 in this study. Red arrows indicate the locations of the variants; black arrows indicate the proband; black symbols indicate patients; white symbols indicate unaffected individuals. Abbreviations: Ref., reference sequence.

**Supplementary Tables**

**Supplementary Table 1.** Primers for Sanger sequencing amplification

| Families | Primer name | Primer sequence (5’-3’) |
| --- | --- | --- |
| Family1 | *CNNM2*-c.890_892delAGG-F | GCTACGCAAGATGGAGAAGA |
| Family1 | *CNNM2*-c.890_892delAGG-R | CGATACCGATGGTGGAGACT |
| Family2 | *CNNM2*-c.1079C>G-F | GCAGGTGATCTTCATTTCGC |
| Family2 | *CNNM2*-c.1079C>G-R | CGGAGCATCTCCAGCAGT |

**Supplementary Table 2.** Worldwide distribution of *CNNM2* variants in probands with HOMGSMR1 as reported

| **Cases** | **Nationality**  **/ Ethnicity** | **Gender** | **Variants** | **Variant type** | **Inheritance** | **ID / DD** | **Seizures** | **Hypomagnesemia** | **Abnormal behaviors** | **Facial features** | **Obesity** | **Other phenotypes** | **Brain MRI** | **ACMG criteria** |
| --- | --- | --- | --- | --- | --- | --- | --- | --- | --- | --- | --- | --- | --- | --- |
| (Panda et al., 2021) | India | F | c.82G>A,  p.A28T | Missense | Het,  *de novo* | Y | Y, refractory | Y | NA | NA | NA | Microcephaly, central hypotonia | No abnormality | NA |
| (Franken et al., 2021) | Caucasian | M | c.143T>C, p.L48P | Missense | Het, inherited from the affected mother | Y, motor skill defects | Y | Y, refractory | N | NA | NA | - | No abnormality | NA |
| (Bosman et al., 2024) | NA | F | c.274G>C, p.A92P | Missense | Het,  *de novo* | Y | N | N | Y | NA | Y | - | NA | LP |
| (Arjona et al., 2014) | Serbia | M | c.364G>A, p.E122K | Missense | Hom | Y | Y | Y | NA | NA | NA | Microcephaly | Myelinization defects, opercularization defect, widened outer cerebrospinal liquor spaces | NA |
| (Arjona et al., 2014) | Serbia | F | c.364G>A, p.E122K | Missense | Hom | Y | Y, refractory | Y, refractory | NA | NA | NA | Microcephaly | NA | NA |
| (Xu et al., 2022) | China | F | c.566A>G, p.Y189C | Missense | Het,  *de novo* | Y | Y | Y | NA | NA | NA | Atrial septal defect | No abnormality | NA |
| (Arjona et al., 2014) | Germany | F | c.806C>G, p.S269W | Missense | Het,  *de novo* | Y | Y, refractory | Y, refractory | NA | NA | Y | - | No abnormality | NA |
| (Zhang et al., 2021) | China | M | c.814T>C, p.F272L | Missense | Het,  *de novo* | Y | Y | Y | NA | NA | NA | - | No abnormality | LP |
| (Liu et al., 2023) | China | M | c.838_843delATGGCC, p.M280_A281del | In-frame deletions | Het,  *de novo* | Y | Y | Y, refractory | NA | NA | NA | - | No abnormality | LP |
| (Franken et al., 2021) | Caucasian | M | c.942C>G, p.Y314X | Nonsense | Het, inherited from the affected mother | Y | N | Y | N | NA | Y | - | No abnormality | NA |
| (Franken et al., 2021) | Sub-Saharan African | M | c.961_963del, p.L321del | In-frame deletions | Het,  *de novo* | Y | Y | Y | N | NA | Y | - | No abnormality | NA |
| (Franken et al., 2021) | Caucasian | M | c.970G>A, p.V324M | Missense | Het,  *de novo* | Y | Y | Y | N | NA | Y | - | No abnormality | NA |
| (Bosman et al., 2024) | NA | M | c.970G>C, p.V324L | Missense | Het,  *de novo* | Y | Y | Y, refractory | Y | NA | Y | - | NA | P |
| (Zhang et al., 2021) | China | F | c.976G>C, p.V326L | Missense | Het,  *de novo* | Y | Y | Y, refractory | NA | NA | NA | - | No abnormality | LP |
| (Arjona et al., 2014) | Poland | F | c.988C>T, p.L330F | Missense | Het,  unknown | Y | Y | Y | NA | NA | NA | Dysesthesia | NA | NA |
| (Franken et al., 2021) | Caucasian | F | c.1016G>A, p.G339D | Missense | Het,  *de novo* | Y | N | Y | NA | NA | N | - | NA | NA |
| (Arjona et al., 2014) | Germany | F | c.1069G>A, p.E357K | Missense | Het,  *de novo* | Y | Y, refractory | Y, refractory | NA | NA | Y | - | No abnormality | NA |
| (Arjona et al., 2014) | Germany | M | c.1069G>A, p.E357K | Missense | Het,  *de novo* | Y | Y, refractory | Y, refractory | Y | NA | NA | - | No abnormality | NA |
| (Franken et al., 2021) | Caucasian | M | c.1094C>T, p.S365F | Missense | Het,  *de novo* | Y | Y | N | N | NA | N | - | Y | NA |
| (Bamhraz et al., 2021) | Canada | F | p.R366P | Missense | Het,  *de novo* | Y | NA | Y, refractory | Y | NA | Y | Anorexia nervosa | No abnormality | NA |
| (Bosman et al., 2024) | NA | NA | c.1147A>G, p.M383V | Missense | Het, inherited from the affected mother | Y | N | Y | Y | NA | N | - | NA | LP |
| (Franken et al., 2021) | Caucasian | F | c.1253T>C, p.L418P | Missense | Het,  *de novo* | Y | Y | Y | N | NA | NA | - | No abnormality | NA |
| (Bosman et al., 2024) | NA | M | c.1310G>A, p.G437E | Missense | Het,  *de novo* | Y, dyslexia | N | Y | Y | NA | N | Multiple thyroid colloid cysts | NA | P |
| (Tseng et al., 2022) | Taiwan, China | F | c.1437G>T, p.R480L | Missense | Het,  *de novo* | Y | Y | Y, refractory | NA | NA | NA | - | NA | NA |
| (Petrakis et al., 2022) | Greece | F | c.1444C>G, p.P482A | Missense | Het,  unknown | N | N | Y | NA | NA | N | Headache, muscle aches, paresthesia of the upper extremities | Two isolated demyelinating-type lesions near  the corpus callosum | LP |
| (Wang et al., 2023) | China | M | c.1448delT, p.V483GfsTer29 | Frameshift | Het,  *de novo* | Y | Y | Y | NA | NA | NA | - | Localized widening of the subarachnoid space. | P |
| (Accogli et al., 2019) | Italy | M | c.1642G>A,p.V548M | Missense | Hom | Y | Y, refractory | Y, refractory | NA | Enlarged nares, thick and uplifted  earlobes | NA | Microcephaly, spastic-dystonic tetraparesis and hyperreflexia,  wrist widening, pectus excavatum, mild  scoliosis with hyperkyphosis, multiple joint contractures | Cerebral cortical atrophy, global reduction of white matter | NA |
| (Li et al., 2021) | China | F | c.2228C>T, p.S743F | Missense | Het,  *de novo* | Y | Y, refractory | Y | Y | NA | N | Funnel-shaped chest | No abnormality | LP |
| (Franken et al., 2021) | Caucasian | F | c.2384C>T, p.S795L | Missense | Het,  *de novo* | Y | N | N | N | NA | N | - | No abnormality | NA |
| (Franken et al., 2021) | Caucasian | F | c.2389C>T, p.R797X | Nonsense | Het,  *de novo* | Y | NA | N | Y | NA | Y | - | Slightly hyperintense white matter located in the corona radiata and centrum semiovale | NA |
| (Franken et al., 2021) | Caucasian | F | Del exon 1-4 | Exon deletions | Het,  *de novo* | Y | Y | Y | N | NA | Y | - | No abnormality | NA |
| (Franken et al., 2021) | Sub-Saharan African | M | Del exon 3-8 | Exon deletions | Het,  ongoing | Y | Y | N | N | NA | N | - | No abnormality | NA |

**Abbreviations:** ID, intellectual disability; DD, developmental delay; EEG, electroencephalogram; MRI, magnetic resonance imaging; ACMG, American College of Medical Genetics and Genomics; F, female; M, male; Het, heterozygous; Hom, homozygous; Del, deletion; Y, yes; N, no; P, pathogenic; LP, likely pathogenic; NA, not applicable; (-), absence.

**References**

Accogli, A., Scala, M., Calcagno, A., Napoli, F., Di Iorgi, N., Arrigo, S., et al. (2019). CNNM2 homozygous mutations cause severe refractory hypomagnesemia, epileptic encephalopathy and brain malformations. *Eur J Med Genet* 62(3)**,** 198-203. doi: 10.1016/j.ejmg.2018.07.014.

Arjona, F.J., de Baaij, J.H., Schlingmann, K.P., Lameris, A.L., van Wijk, E., Flik, G., et al. (2014). CNNM2 mutations cause impaired brain development and seizures in patients with hypomagnesemia. *PLoS Genet* 10(4)**,** e1004267. doi: 10.1371/journal.pgen.1004267.

Bamhraz, A.A., Franken, G.A.C., de Baaij, J.H.F., Rodrigues, A., Grady, R., Deveau, S., et al. (2021). Diagnostic Dilemma in an Adolescent Girl with an Eating Disorder, Intellectual Disability, and Hypomagnesemia. *Nephron* 145(6)**,** 717-720. doi: 10.1159/000518173.

Bosman, W., Franken, G.A.C., de Las Heras, J., Madariaga, L., Barakat, T.S., Oostenbrink, R., et al. (2024). Hypomagnesaemia with varying degrees of extrarenal symptoms as a consequence of heterozygous CNNM2 variants. *Sci Rep* 14(1)**,** 6917. doi: 10.1038/s41598-024-57061-7.

Franken, G.A.C., Müller, D., Mignot, C., Keren, B., Lévy, J., Tabet, A.C., et al. (2021). The phenotypic and genetic spectrum of patients with heterozygous mutations in cyclin M2 (CNNM2). *Hum Mutat* 42(4)**,** 473-486. doi: 10.1002/humu.24182.

Li, X., Bao, S., Wang, W., Shi, X., Hu, Y., Li, F., et al. (2021). Case Report: CNNM2 Mutations Cause Damaged Brain Development and Intractable Epilepsy in a Patient Without Hypomagnesemia. *Front Genet* 12**,** 705734. doi: 10.3389/fgene.2021.705734.

Liu, C.X., Zhang, Y., Hu, L., and Q.M., H. (2023). Hypomagnesemia, seizures, mental retardation caused by heterozygous mutation of CNNM2: a case report and literatures review. *Journal of Chinese Physician* 25(12)**,** 1781-1784. doi: 10.3760/cma.j.cn431274-20231105-00511.

Panda, P.K., Lourembam, R., and Sharawat, I.K. (2021). CNNM2 Heterozygous Variant Presenting as Hypomagnesemia and West Syndrome: Expanding the Spectrum of CNNM2 Gene-Related Epileptic Disorders. *Ann Indian Acad Neurol* 24(5)**,** 781-783. doi: 10.4103/aian.AIAN_1130_20.

Petrakis, I., Drosataki, E., Stavrakaki, I., Dermitzaki, K., Lygerou, D., Konidaki, M., et al. (2022). The p.Pro482Ala Variant in the CNNM2 Gene Causes Severe Hypomagnesemia Amenable to Treatment with Spironolactone. *Int J Mol Sci* 23(13)**,** 7284. doi: 10.3390/ijms23137284.

Tseng, M.H., Yang, S.S., Sung, C.C., Ding, J.J., Hsu, Y.J., Chu, S.M., et al. (2022). Novel CNNM2 Mutation Responsible for Autosomal-Dominant Hypomagnesemia With Seizure. *Front Genet* 13**,** 875013. doi: 10.3389/fgene.2022.875013.

Wang, L., Zhang, H., Luo, J., Qi, F., Liu, Y., Zhang, K., et al. (2023). Analysis of CNNM2 gene variant in a child with Hypomagnesemia, seizures, and mental retardation syndrome. *Zhonghua yixue yichuanxue zazhi* 40(8)**,** 1004-1008. doi: 10.3760/cma.j.cn511374-20220915-00622.

Xu, X., Hou, S., Sun, W., Zhu, J., Yuan, J., Cui, Z., et al. (2022). Rare hypomagnesemia, seizures, and mental retardation in a 4-month-old patient caused by novel CNNM2 mutation Tyr189Cys: Genetic analysis and review. *Mol Genet Genomic Med* 10(4)**,** e1898. doi: 10.1002/mgg3.1898.

Zhang, H., Wu, Y., and Jiang, Y. (2021). CNNM2-Related Disorders: Phenotype and Its Severity Were Associated With the Mode of Inheritance. *Front Pediatr* 9**,** 699568. doi: 10.3389/fped.2021.699568.
